# A Rare Case of Complicated Kommerell's Diverticulum in a Middle‐Aged Woman

**DOI:** 10.1002/ccr3.72708

**Published:** 2026-05-13

**Authors:** Elham khorasani, Hourieh Soleimani

**Affiliations:** ^1^ Department of Internal Medicine, Faculty of Medicine Mashhad University of Medical Sciences Mashhad Iran; ^2^ Department of Radiology, Faculty of Medicine Mashhad University of Medical Sciences Mashhad Iran

**Keywords:** aneurysm, angiography, cardiovascular, computed tomography, Kommerell's diverticulum

## Abstract

Kommerell's Diverticulum is a rare vascular anomaly that can manifest as an anterior mediastinal mass. Considering its rarity and possible lethal complications, it is crucial to consider this abnormality when dealing with anterior mediastinal vascular lesions. Computed tomography (CT) angiography provides valuable information about this anomaly and its complications.

## 1 Introduction

Kommerell's Diverticulum (KD), a rare congenital anomaly of the aortic arch, was first described by Burkhard Kommerell in 1936. He observed a pulsatile mass exerting pressure on the esophagus during a barium swallow [1]. This anomaly results from a defect in the organogenesis of the primitive aortic arches and the failure of regression of the dorsal fourth aortic arch. The term “diverticulum” refers to a localized, conical dilatation of the aorta near the origin of an aberrant subclavian artery [2].

Although most patients with KD are asymptomatic and diagnosed incidentally, the anomalous course of this artery and its compressive effect on mediastinal structures and adjacent organs, such as the trachea and esophagus, can lead to a wide range of clinical symptoms. These symptoms include cough, wheezing, dyspnea, back pain, and dysphagia [3, 4, 5, 6]. The prevalence of this anomaly in the adult population is reported to be approximately 0.05%–0.1% [7].

## 2 Case Presentation

A 72‐year‐old female, with a history of hypertension (under medication) and a history of baking bread, presented to the emergency department via EMS with chief complaints of dyspnea, cough, and elevated blood pressure. The patient reported dyspnea for approximately 20 years, which was initially classified as NYHA functional class II‐III, but had recently progressed to class IV. She had a history of two previous hospitalizations due to cerebrovascular accident (CVA), the last occurring in the previous year.

The patient denied syncope, dysphagia, or weight loss but reported a history of hypertensive crises (hypertensive urgency/emergency) in her past medical history (PMH).

She also complained of discomfort and diffuse pain in her back and left hemithorax. Her cough was exacerbated by laughing. Upon physical examination, lung auscultation revealed diffuse expiratory wheezing. Her vital signs on admission were blood pressure 130/80 mmHg, heart rate 80 bpm, and oxygen saturation < 90%.

## 3 Investigations

The patient initially presented with respiratory symptoms and immediately underwent chest x‐ray and aortic CTA due to the CXR findings. The patient was referred to a cardiac surgeon and was admitted to the hospital 3 days later. She was planning to undergo open surgical repair and Bentall procedure; however, it was postponed due to the inability to find the necessary surgical equipment. Four weeks later, after partial improvement of symptoms, the patient was discharged and 1 week after discharge, corresponding to 5 weeks after the initial CT scan, she was readmitted urgently because of worsening shortness of breath and chest pain. In this admission, a repeat CT scan was performed and the patient underwent open surgical repair. Endovascular device unavailability, relatively large size of aneurysm, and synchronous aortic arch aneurysm were factors influencing the treatment strategy.

**FIGURE 1:**
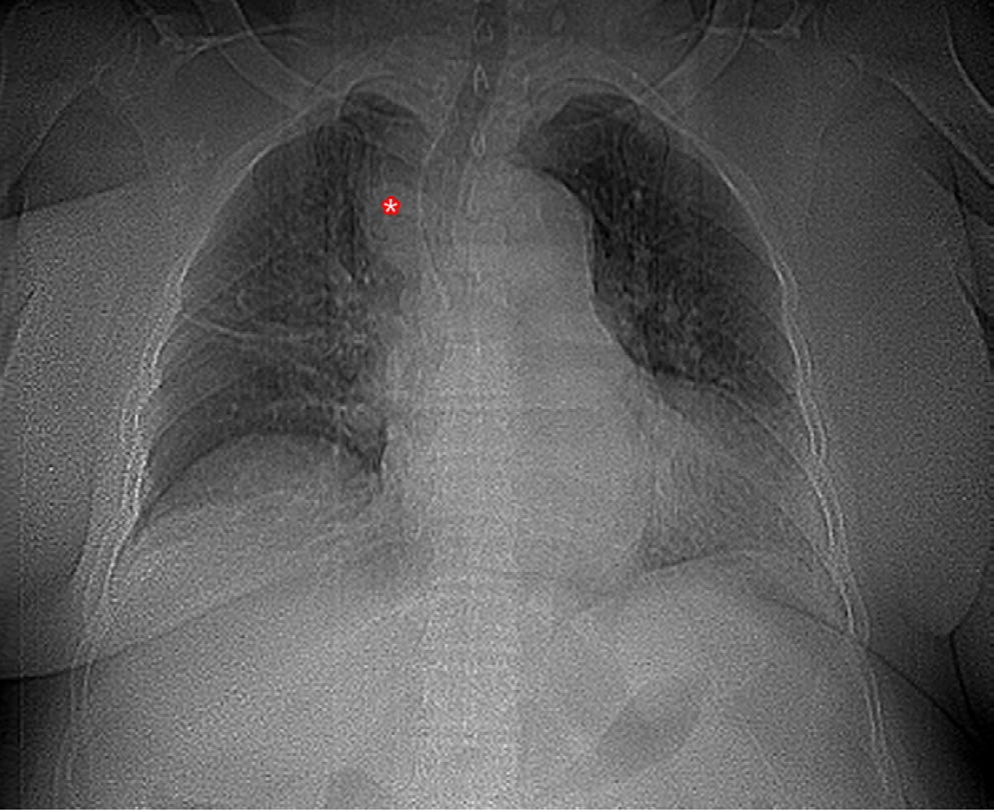
Scout view of CTA at the patient's initial presentation, reveals a smooth right paratracheal opacity (*) and dilated aortic arch.

At the first visit, the initial workup, including chest radiography, revealed mediastinal widening with a well‐defined oval opacity at the right paratracheal region (Figure 1). Lung parenchyma and pulmonary vascular patterns were reported as normal. To further evaluate the mediastinal vascular pattern, additional imaging, including computed tomography angiography (CTA) of the chest, was performed.

CT scan was performed using a 16‐slice multi‐detector CT (MDCT) scanner. Axial images were performed with slice thickness of 2.5‐mm, 1.5 pitch, 120 kVp voltage. Imaging was obtained 35 s after intravenous administration of 120 mL of Visipaque (320 mg/mL) at a rate of 5 mL/s through an antecubital vein. CT images were acquired with coronal and sagittal reconstructions using vascular/heart window settings.

**FIGURE 2:**
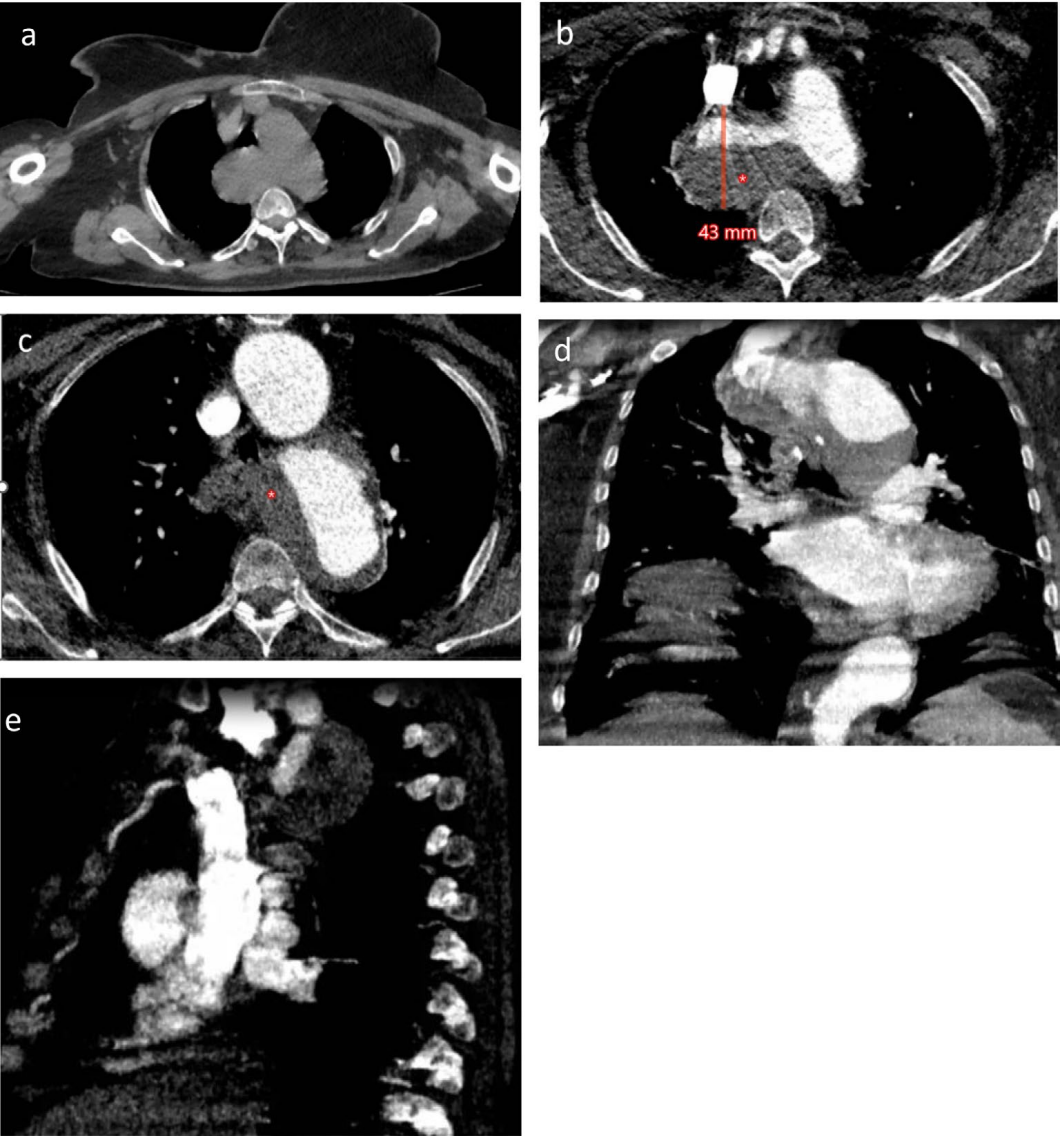
Thoracic aorta CTA at the patient's initial presentation. (a) Non‐contrast axial CT scan under soft tissue/mediastinal window settings, shows an aneurysmal dilation of the origin of ARSA. (b, c) Axial images of CT angiography (arterial phase) using vascular/heart window settings, show Kommerell aneurysm, with a maximum diameter of 43 mm, contains mural thrombus (*) and a dilated aortic arch. (d) Coronal reconstruction of arterial phase image under vascular/heart window settings, reveals the mural thrombus (*) in both the Kommerell aneurysm and the aortic arch. (e) Coronal reconstruction of arterial phase images using vascular/heart window settings shows a cross‐sectional view of ARSA and Kommerell aneurysm.

CTA revealed a left‐sided aortic arch with an aberrant right subclavian artery (ARSA). A round aneurysmal dilatation was seen at the origin of ARSA, with a maximum diameter of 4.3 cm, compatible with kommerell diverticulum. This vascular lesion exerts compression on the posterior wall of the trachea. A mural thrombus was identified in the aneurysm. The aortic arch measured 5.8 cm and contained mural thrombus (Figure 2).

Review of the patient's medical records indicated that a CT scan performed 2 years prior had also demonstrated evidence of KD with similar characteristics, without any complications during this period; however, KD had increased in size by 5 mm compared with the prior CT scan (measuring 3.8 cm in CT performed 2 years earlier).

Cardiac evaluations, including echocardiography, showed no evidence of pulmonary hypertension and an ejection fraction (EF) of 55%. A dilated ascending aorta with a diameter of 4.7 cm was also reported. Electrocardiogram (ECG) showed axis: −30‐degree axis and prominent S in V5–V6 lead. Initial laboratory tests, including complete blood count (CBC) and electrolytes, showed no significant abnormalities. Troponin levels were negative.

Initial conservative management included blood pressure control, oxygen therapy, and respiratory support. After diagnostic workup and initial symptom management, the patient was referred to a cardiac surgeon, admitted to the hospital, and planned for definite surgical treatment. Although it could not be performed because of device unavailability. The patient was discharged and was readmitted after her respiratory symptoms had progressed. Transesophageal echocardiography (TEE) and conventional angiography were performed, and CT angiography was repeated.

**FIGURE 3:**
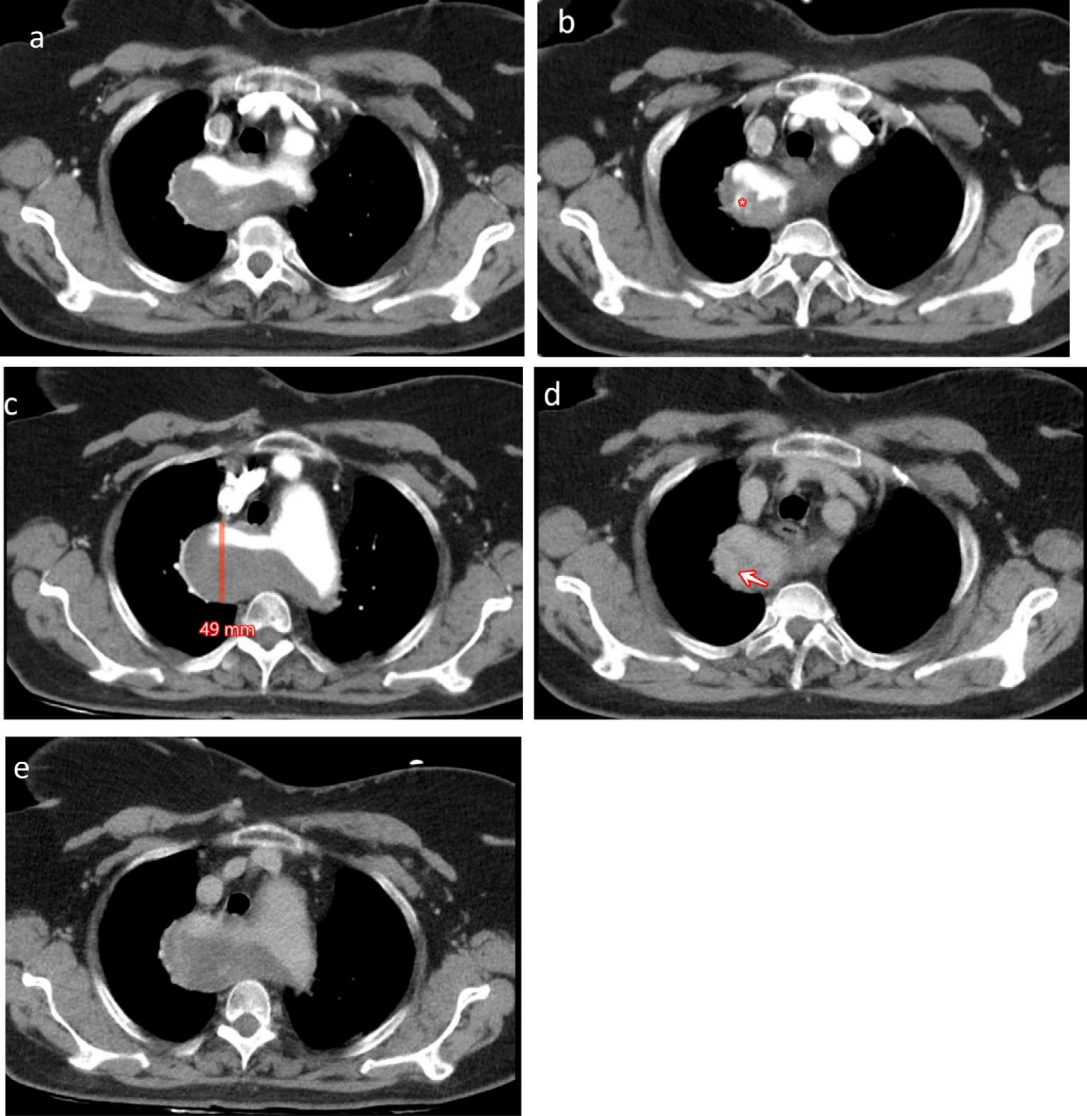
The 5‐week follow‐up CT scan of the same patient. (a–c) The arterial phase (CTA) axial images under soft tissue/mediastinal window settings, reveal the Kommerell aneurysm with a maximum diameter of 49 mm, irregularity of the inner wall of ARSA, and a hyperdense crescent sign in the mural thrombus (*). (d, e) The delayed phase images (7 min), show increased attenuation of the mural thrombus (arrow).

The second CTA showed a 6 mm increase in the maximum diameter of KD, reaching 49 mm, and was associated with a hyperdense crescent sign within the mural thrombus (Figure 3). Irregularity of the enhancing vascular lumen, hyperdense contrast leak into the mural thrombus, and increased attenuation of the thrombus were new findings compared with the initial CTA. These findings were red flags for “impending rupture” state of the aneurysm [8]. Therefore, the patient underwent open surgical repair via median sternotomy 7 days after admission. After systemic heparinization, cardiopulmonary bypass was initiated using arterial cannulation of the brachiocephalic artery and two‐stage right atrial venous cannulation. The surgical team initiated systemic cooling to achieve the target temperature of 22°C. Aortic cross‐clamping and infusion of cold blood cardioplegia were administered resulting in cardiac arrest. The ascending aorta was opened, and a severely calcified aortic valve was visualized. A large aneurysm of the ascending aorta was resected and replaced with a new Dacron tube graft. The patient was successfully weaned from cardiopulmonary bypass and achieved hemostasis. She was admitted to the Intensive Care Unit (ICU) without immediate postoperative complications; however, she died 3 days later due to postoperative cardiovascular complications.

## 4 Discussion

In normal vascular anatomy, the left subclavian artery directly originates from the aortic arch, while the right subclavian artery branches from the brachiocephalic artery. These arteries are responsible for supplying blood to the upper extremities, chest, and brain [9]. The typical vascular anatomy of the human aortic arch results from the regression of the right fourth embryonic aortic arch and the persistence of the left fourth embryonic aortic arch. Kommerell Diverticulum (KD), as a developmental anomaly of the fourth aortic arch system, arises from the persistence of the embryonic right fourth aortic arch [10, 11]. Although often asymptomatic and incidentally discovered in adulthood, KD is clinically significant due to its potential for serious complications.

The prevalence of symptoms in KD patients varies. A study by Poterucha et al. (2015) [12] reported symptomatic patients in approximately 61% of cases [12], indicating a significant proportion may present clinical complaints. In our patient's case, symptoms such as exertional dyspnea, cough, and wheezing could initially have been attributed to more common underlying conditions, such as chronic obstructive pulmonary disease (COPD). However, given the diagnosis of Kommerell Diverticulum and its mass effect on adjacent structures, some of her symptoms can be linked to this vascular anomaly. This case underscores the importance of clinical suspicion in differentiating KD‐related symptoms from other common causes. In many instances, KD symptoms may be overlooked in favor of other diagnoses, and the connection between symptoms and the vascular anomaly is only recognized retrospectively.

The clinical significance of Kommerell Diverticulum extends beyond compressive symptoms due to its high potential for serious vascular complications, including aneurysm rupture and aortic dissection. Studies have shown that the significant risk of these complications [2, 10]. Given these potentially life‐threatening complications, educating patients about the nature of their condition and its possible outcomes is of particular importance [4]. Furthermore, regular follow‐up for asymptomatic patients is strongly recommended to monitor the size and characteristics of the KD and to detect any potential changes or complications early. While no specific, unified treatment protocol for KD exists, factors such as aneurysm size, patient age, and treatment compliance are considered important in its management.

Hyperdense crescent sign represents the accumulation of crescent‐shaped blood in the mural thrombus of an enlarged aneurysm and was initially described by Mehard et al. [13] This important sign indicates the presence of fresh blood within the mural thrombus and can be observed on both non‐enhanced and contrast‐enhanced CT scans. It is a marker of aneurysm instability and is associated with an increased risk of aneurysm rupture [8].

Therapeutic interventions are indicated for symptomatic or complicated patients. The treatment strategy should be individualized. Watch and waiting, open surgery, endovascular treatment, and hybrid techniques are considered therapeutic options. Conservative management is not a good option for this patient due to enlarging KD, aneurysmal dilation of the aortic curve, and the presence of related symptoms. Endovascular repair is increasingly used and preferred over open surgical repair; however, vascular anatomy complexity, treatment risk assessment, and device availability are important factors [14]. Therefore, careful perioperative assessment through multidisciplinary management is critical for selecting the best treatment strategy. Open surgical repair remains the preferred option in complicated aortic aneurysms; rare vascular anomalies and endovascular options are limited [15, 16].

## 5 Conclusion

An aberrant right subclavian artery (ARSA), also known as Arteria Lusoria, although rare, is the most common congenital anomaly of the aortic arch. This artery is associated with various vascular anomalies, of which Kommerell's Diverticulum is one. KD occurs as an aneurysmal dilatation at the origin of an aberrant subclavian artery, which may be on the right or left side of the aortic arch [7, 11]. Although often asymptomatic and diagnosed incidentally, it can be associated with a wide range of clinical symptoms due to its compressive effect on adjacent structures. This case report emphasizes the importance of timely diagnosis and differentiation of its symptoms from other conditions, even in cases where initial symptoms are attributed to more common causes. Given the high potential of this anomaly for serious vascular complications such as aneurysmal rupture and aortic dissection, patient education and regular periodic follow‐up, even in asymptomatic cases, are of significant clinical importance. These measures are essential for preventing life‐threatening complications and optimizing the management of patients with Kommerell's Diverticulum.

## Author Contributions

**Elham khorasani:** investigation, project administration, writing – original draft. **Hourieh Soleimani:** conceptualization, investigation, writing – review and editing.

## Funding

The authors have nothing to report.

## Consent

Written informed consent was obtained from the patient for publication of this case report and any accompanying images.

## Data Availability

The data that support the findings of this study are available from the corresponding author upon reasonable request. The data are not publicly available due to privacy and ethical restrictions.
